# Short Course of Systemic Steroids for Acute Respiratory Diseases During Infancy and Final Adult Height, Weight, and BMI: Preliminary Results from a Prospective Cohort Study

**DOI:** 10.3390/jcm14020387

**Published:** 2025-01-09

**Authors:** Athina Papadopoulou, Stavroula Lampidi, Konstantinos Makris, Efstathios Chronopoulos

**Affiliations:** 1 Pediatric Allergy and Asthma Unit, KAT General Hospital, 14561 Athens, Greece; stavroul.lab@gmail.com; 2 Clinical Biochemistry Department, KAT General Hospital, 14561 Athens, Greece; kostas.makris.km@gmail.com; 3 Laboratory of Research of the Musculosceletal System, National and Kapodistrian University of Athens, KAT General Hospital, 14561 Athens, Greece; stathi24@yahoo.gr

**Keywords:** systemic steroids, infancy, acute respiratory disease, final height, BMI

## Abstract

**Background:** Systemic corticosteroids are frequently used to manage acute respiratory diseases in infancy, but concerns about the long-term impacts on growth remain. This study aimed to evaluate the impact of short courses of systemic steroids administered exclusively during infancy on final adult height, weight, and BMI, adjusted by sex and cumulative steroid use. **Methods**: A prospective cohort study was conducted including 257 participants (49.4% males, 11.2 ± 3.5 years) of which two groups of cases were firstly analyzed: the control group (CG) and the group that received systemic steroids only during infancy (ssccINF). Final adult height, weight, and BMI were compared between the groups, adjusted also for breastfeeding history, food allergies, history of fractures, physical activity, and family smoking habits. **Results:** No significant differences in final adult height were observed between males in the CG and ssccINF group (179.32 vs. 179.40). In females, the ssccINF group was slightly shorter by 2.5 cm (165.51 vs. 162.98), although this difference was not linked to cumulative days of steroid use during infancy (mean = 3.91 ± 2.37, *p* = 0.37). A regression analysis revealed no significant influence of additional covariates on height, weight, or BMI outcomes. **Conclusions:** Short courses of systemic steroids administered exclusively during infancy did not appear to have a significant long-term impact on growth. The minor height difference observed in females was not associated with steroid use duration. These findings suggest that the benefits of short-term steroid therapy, such as reduced hospitalizations and improved management of acute respiratory diseases, outweigh potential risks, supporting its safe use in clinical practice.

## 1. Introduction

Systemic corticosteroids are the cornerstone of effective treatment for many common emergent pediatric respiratory conditions, including croup, bronchiolitis, and asthma attack, in otherwise healthy children [1,2,3,4]. While long courses of steroids or recurrent use have been associated with many adverse events (AEs), such as growth retardation, adrenal insufficiency, diabetes mellitus, blood pressure, psychosis, osteopenia, and fractures [5,6,7], the use of short, singular courses is considered uneventful. Even though reviews have emphasized the inconsistencies in definitions and assessments, as well as the variations in corticosteroid formulations and dosages among studies, they suggest that the use of a few short-term, high-dose systemic corticosteroids courses is not associated with an increase in AEs across organ systems [3,4]. However, rare temporal immediate adverse events have been reported, but uncertainties remain, particularly regarding their long-term impacts on growth outcome and bone density [8,9].

In adults, it is common for asthmatic patients to receive at least one short oral course of corticosteroids annually for acute exacerbations [10,11]. Studies have demonstrated that even a short-term oral/systemic corticosteroid course (SSCC) is associated with an increased risk of both acute and chronic AEs, even when doses are relatively low [12]. Growing evidence suggests that even brief dosing periods (3–7 days) of an SSCC are enough to cause significantly loss of bone density [13]. Similarly, data from US insurance claims including approximately 1.5 million people show that an SSCC was associated with an increased risk of fracture (RR = 1.9) within the first month after initiation, compared with therapies without steroids [14]

Similar findings have been observed in children. The use of glycocorticoids, particularly in repeated cycles at high cumulative doses, has been shown to decrease bone density and increase the risk of fractures [8,9,15,16]. In children with rheumatic diseases or severe uncontrolled asthma, growth retardation has been attributed not only to chronic inflammation but also to systemic steroid therapy [16,17]. In contrast, the effects of inhaled corticosteroids (ICS) on growth have been extensively studied, although the results have been inconsistent. Most studies indicate that ICS use does not significantly affect growth during infancy [18,19,20]. However, a small but consistent reduction in growth velocity is observed during childhood, with more pronounced delays during puberty. Finally, by age 19, most children achieve normal adult height, despite delayed peak height velocity during adolescence, particularly in asthmatic boys. However, recent reports suggest that the initial reduction in height observed in prepubertal children treated with ICS persists into adulthood as a slight decrease in final adult height, although the reduction is neither progressive nor cumulative [21,22]. While regular use of ICS may cause a small reduction in linear growth in children with asthma, the benefits of controlling asthma generally outweigh the potential adverse effects on growth [23].

On the other hand, the impact of short, singular courses of systemic corticosteroids on the growth of otherwise healthy children treated for acute respiratory conditions remains unclear. Given the increasing use of systemic corticosteroids in preschool-aged children and the considerable uncertainty faced by clinicians, there is an urgent need for studies evaluating the long-term effects of SSCCs on linear growth and final adult height.

The aim of this study was to retrospectively (from birth until the age of entry in the study) and prospectively (from the entry time until early adulthood) assess the use of systemic corticosteroids and to follow the growth trajectory of otherwise healthy preschool-aged children until early adulthood. Specifically, the study seeks to determine whether short courses of systemic corticosteroids administered only during infancy for acute respiratory conditions influence final adult height, weight, and BMI in this population. This focus on the long-term effects of early-life systemic corticosteroid use on final growth provides a novel contribution to the field.

## 2. Patients and Methods

### 2.1. Study Design

This is a prospective, observational cohort and single-center study conducted at the Pediatric and Adolescent Outpatient Unit of a tertiary General Hospital. Data were collected from the birth records of children who visited the unit for primary or secondary care between January 2012 and December 2014. Children were followed up with regular visits until adulthood.

### 2.2. Study Sample and Data Collection

Healthy children visiting the unit for primary care (e.g., vaccinations and annual check-ups) were invited to participate in the study. Upon obtaining written informed consent, a total of 295 subjects (mean age = 11.2 ± 3.5 years) were enrolled.

Retrospective data were collected from medical records. Prospective data were collected via annual clinic visits, parental interviews, and medical records until the children reached 20 years of age. Telephone contact was made every six months to gather interim medical information and remind parents of upcoming visits.

Data collection focused on the children’s health and medication use during various life stages, as follows: gestational and neonatal period; infancy (<2 years old); preschool years (2–5 years old); school years (5.1–12 years old); and adolescence (>12 years old)

**Figure 1:**
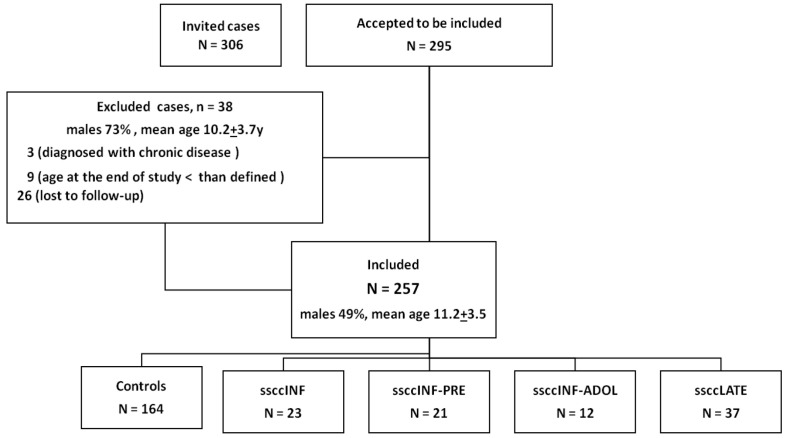
Flowchart of included and excluded cases. Overall, 257 subjects were included and divided into the following 5 groups based on the longest duration of systemic steroids use: control group (CG)—children who had never been prescribed systemic steroids; SSCC-INF group—children who used an SSCC during infancy (<2 years old) and did not require corticosteroid therapy afterward; SSCC-INF-PRE group—children who used an SSCC during infancy and preschool years; SSCC-INF-ADOL group—children who used an SSCC from infancy up to adolescence; SSCC-LATE group—children who used an SSCC after infancy.

Particular attention was given to the type, dosage, and route of steroid administration. Children born preterm or those with chronic diseases (except asthma) were excluded. During follow-up, three children were excluded because of the development of new chronic diseases (celiac disease, inflammatory bowel disease, and severe trauma), and nine boys were excluded as they had not yet reached their final height (Figure 1).

Subjects were categorized into the following five groups based on their systemic corticosteroid (SSCC) use in an attempt to better analysis the long-term effects:

Control group (CG): children who had never been prescribed systemic steroids;

SSCC-INF group: children who used an SSCC during infancy (<2 years old) and did not require corticosteroid therapy afterward;

SSCC-INF-PRE group: children who used an SSCC during infancy and preschool years;

SSCC-INF-ADOL group: children who used an SSCC from infancy up to adolescence.

SSCC-LATE group: children who used an SSCC after infancy;

For this preliminary analysis, only the control group and the SSCC-INF group were included.

### 2.3. Measurements

At the time of inclusion, historical data were obtained from medical records. Thereafter, trained nurses measured the children’s anthropometric characteristics annually during scheduled follow-up visits until adulthood. For children > 2 years old, standing height was always measured with a Seca Minimeter (Seca Equipment Limited, Northants), after they removed their shoes, and body weight with a Seca weighing scale, ensuring they were dressed in light clothing (Seca, MD 21076 Hanover, NH, USA).

Final adult height was defined as the height measured at or after the age of 17 years for females and 19 years for males. Missing data from medical records or during the 15-year follow-up were noted in approximately 50% of cases. However, height and weight measurements at the time of inclusion and at final follow-up were available for 100% and 90% of the subjects respectively (Figure 1).

### 2.4. Ethics

This study adhered to the principles outlined in the Declaration of Helsinki and was approved by the Ethics Committee of the Hospital. Written informed consent was obtained from all participants’ parents or legal guardians.

### 2.5. Statistics

Statistical analyses were performed using SPSS version 25.0 for Windows (IBM Hellas Inc., New York, NY, USA). A power analysis indicated that the sample size was sufficient to achieve a statistical power of 95% for detecting a 0.5 cm annual height change, with a 5% significance level for two-sided hypotheses.

For the descriptive statistics, continuous variables are reported as the mean with standard deviation (SD) or median with interquartile range (IQR), and categorical variables are presented as counts and percentages. For group comparisons, Bayesian statistics were employed, using Pearson correlation for categorical variables and k-independent tests for continuous variables, to assess baseline differences among the groups. Basic demographic characteristics of patients lost to follow-up were compared with those of included cases.

For the preliminary analysis, subjects from the control group and SSCC-INF group were analyzed. Final height at early adulthood was the primary outcome. Using a longitudinal analysis repeated measurements, the changes in height that occur until the age of 20 years in relation to steroids use during this period of time were reflected. Group comparisons of final height were adjusted for sex. Using a multiple linear regression model, additional covariates included age at entry, breastfeeding history, food allergies, lifetime asthma, history of fractures, regular physical activity, and family smoking habits were evaluated as fixed variables.

The adjusted mean height in each study group was computed from the regression model at the mean values for the covariates and 95% CI. In addition, a steroids use analysis was performed. Final height was compared according to the cumulative days of systemic corticosteroids using Bayesian statistics, with paired sample t-tests used for parameter estimation. The effect of missing data was addressed using multivariate multiple imputation, generating 13 imputations from simulations of a Bayesian posterior predictive distribution. An iterative Markov chain Monte Carlo (MCMC) method was used to account for missing values. The level of significance was set at *p* < 0.05.

## 3. Results

A total of 257 cases were included in the study, with 49.4% males and a mean age of 11.2 ± 3.5 years. No significant differences were observed between included and excluded cases (Table 1). The control group (CG) comprised 164 subjects (63.8%), while the short course of systemic steroids in infancy group (ssccINF) consisted of 23 subjects (8.9%). A detailed comparison of the demographic and clinical characteristics of the groups is presented in Table 1, highlighting the similarities and ensuring comparability across the cohorts. The ssccINF group used steroids for bronchiolitis in 78.3% of cases and for croup in 21.7%. Additionally, one-quarter of the ssccINF group required hospital admission.

**Figure 2:**
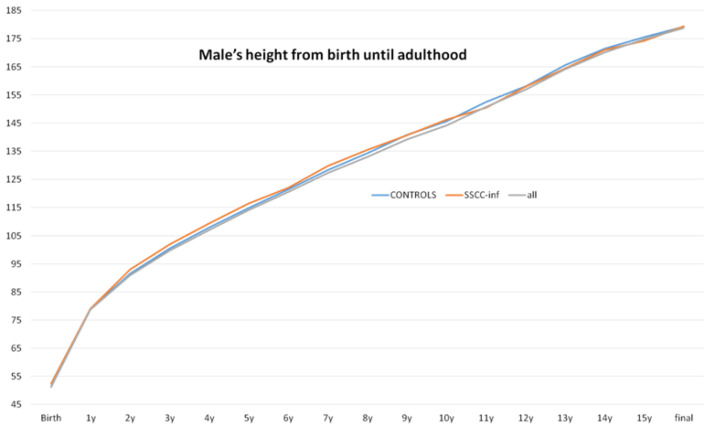
Males’ heights from birth to adulthood. No significant difference was found between the control (never used systemic steroids) and SSCC-INF (children who used an SSCC during infancy (<2 years old) and did not require corticosteroid therapy afterward) groups, *p* = 0.95.

**Figure 3:**
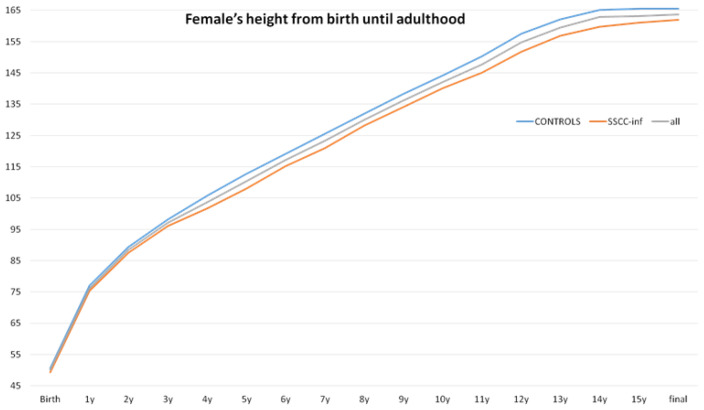
Females’ heights from birth until adulthood. No significant difference was found between the control (never used systemic steroids) and SSCC-INF (children who used an SSCC during infancy (<2 years old) and did not require corticosteroid therapy afterward) groups, *p* = 0.09.

**Figure 4:**
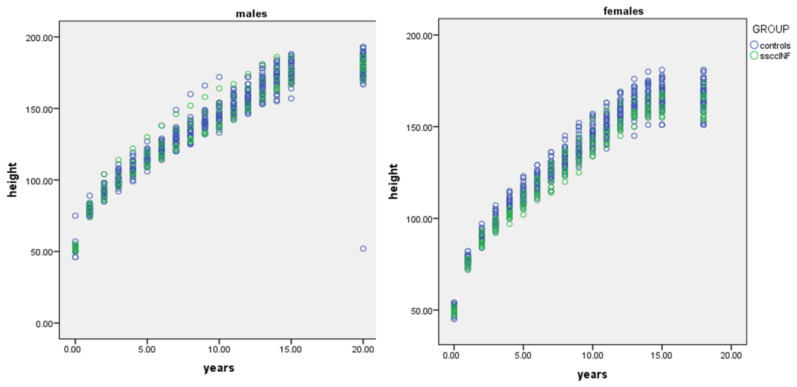
Males’ and females’ heights in a longitudinal scatter model.

Final adult height was evaluated separately by sex. Among males, no significant difference in final height was observed between the groups, with the CG averaging 179.32 cm and the ssccINF group averaging 179.41 cm, *p* = 0.95 (Figure 2). In females, the final height in the CG was 165.51 cm, while in the ssccINF group it was slightly lower, at 162 cm, *p* = 0.09 (Figure 3). Missing interval values, accounted for using the Markov chain Monte Carlo method, did not influence the final outcomes (Figure 4). These results indicate no substantial impact of early systemic steroid use on final height in males, with a minor difference noted in females.

Final weight and BMI was evaluated separately by sex. Among males, no significant difference in final weight and BMI was observed between the groups, with the CG averaging 75.74 kg and 23.59 m^2^/kg and the ssccINF group averaging 72.16 kg and 22.46 m^2^/kg, *p* = 0.31, respectively. In females, the final weight and BMI in the CG were 61.05 kg and 22.29 m^2^/kg, and in the ssccINF group they were 60.27 kg and 22.34 m^2^/kg, *p* = 0.65, respectively.

A multiple linear regression model was used, adjusted by sex, to evaluate additional covariates, including age at entry, breastfeeding history, food allergies, lifetime asthma, history of fractures, physical activity, and family smoking habits. The analysis revealed that none of these factors emerged as significant cofactors influencing final adult height. This suggests that the primary outcome—final height—was not significantly affected by these variables in the context of early systemic steroid exposure (Table 2).

**Figure 5:**
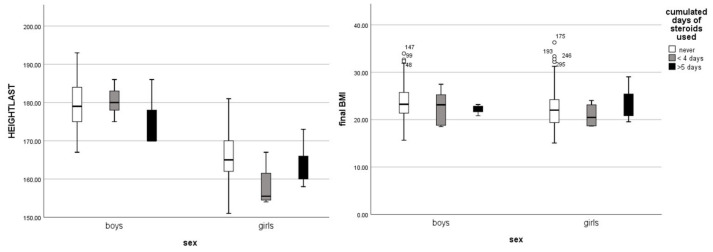
The influence of the cumulative days of steroids use during infancy on final height (*p* = 0.72) and BMI (*p* = 0.8), adjusted by sex.

The cumulative days of steroid use during infancy were further analyzed, with steroids administered either orally or intravenously, primarily for viral laryngeal infections and bronchiolitis. (mean = 3.91 ± 2.37). Males had a mean total steroid use of 3.5 ± 1.72 days, while females used steroids for 4.3 ± 2.8 days, *p* = 0.65. Notably, the total duration of steroid use was not associated with the number of hospitalizations. Furthermore, no significant influence of steroid use on final adult height was observed for either sex, indicating that the short course of systemic steroids did not have a measurable impact on growth outcomes (Figure 5).

As secondary outcomes, weight and BMI were evaluated between the groups adjusted by sex and cumulated days of steroids use. The analysis revealed that none of these factors emerged as significant cofactors influencing final adult weight or BMI, and no significant difference was found between the groups as shown in Table 2 These results suggest that early steroid use did not affect long-term weight or BMI outcomes.

## 4. Discussion

This prospective cohort study found that a short course of systemic steroids only during infancy did not significantly affect final adult height in males or females, nor did it influence weight or BMI in either sex. These findings suggest that short-term steroid use in infancy may have limited long-term impacts on physical growth and body composition.

While no significant difference was observed in the final height of males between the control and ssccINF groups, females in the ssccINF group were found to be slightly shorter—by 2.5 cm—compared to those in the control group. However, this difference was not linked to the cumulative days of steroid use, even though females in the ssccINF group reported using steroids for one more day on average. This increased use of systemic steroids in girls was also not related to any other cofactor, further supporting the limited long-term impact and providing reassurance that brief, early-life systemic steroid use does not appear to adversely impact adult growth outcomes.

Previous studies have reported potential growth suppression in children receiving long-term or high-dose systemic steroids, particularly in those with chronic conditions such as inflammatory disease or severe asthma [5,6,7]. On the other hand, evidence suggests that short-term, high-dose inhaled or systemic corticosteroids use is not associated with an increase in adverse events across organ systems [4]. However, uncertainties remain, particularly regarding recurrent use and growth outcomes. Our study contributes to addressing this gap, and our finding are consistent with research suggesting that short-term use, as in the present cohort, does not result in significant long-term growth impairment. In a review by Harding T. et al., short courses of oral steroids (less than two weeks) in children are very unlikely to cause long-term side effects. On the other hand, children requiring courses for longer than two weeks warrant specialist referral and a weaning plan to reduce adrenal suppression and insufficiency [24].

The use of systemic corticosteroids for long periods is known to affect growth via suppression of the hypothalamic–pituitary–adrenal axis, reducing growth hormone secretion and impairing bone formation [16]. A dose-dependent reduction in bone mineral accretion, as well as an increased risk of osteopenia, has been documented in boys following repeated exposure to short courses of oral steroids over several years. In contrast, this effect was not observed in girls [25]. Additionally, systemic steroid use has been linked to an increased risk of pediatric fractures [15]. However, final height has not been evaluated. The cases in our cohort underwent short durations of steroid exposure, with a mean usage of 3–4 days only during infancy, likely limiting these effects and explaining the lack of significant differences in final height. Despite these concerns, the minimal loss in final height observed in females in this study must be weighed against the well-established benefits of steroid therapy, which include a reduction in hospital admissions and decreased severity of respiratory infection, improving quality of life. These therapeutic advantages underscore the importance of carefully balancing the risks and benefits when prescribing systemic steroids in infancy.

The absence of significant differences in weight and BMI between the groups in our study further reinforces the notion that short-term systemic steroid use does not negatively impact long-term metabolic outcomes or obesity, supporting its safety for short-term use in infancy

Systemic corticosteroids are the cornerstone of effective treatment for many common emergent pediatric respiratory conditions including croup, bronchiolitis, and asthma attack in otherwise healthy children [1,2,3,4]. According to our findings, 8.9% of children in the cohort received short courses of systemic corticosteroids (SSCCs) exclusively during infancy, which appears to have contributed to preventing hospital admissions. Only 26% of these cases were finally admitted to hospital. Moreover, a recent systematic review and meta-analysis concluded that corticosteroid use in the treatment of respiratory diseases in children can significantly shorten hospitalization times and increase the cure rate with transient immediate adverse reactions [3,7]. For children, such as those in our group, who largely had histories of transient wheeze or viral infections during infancy and did not present with severe respiratory disease later in life, our results are clinically important and provide reassurance to pediatricians that short-term systemic steroids used to treat respiratory conditions during infancy are unlikely to have adverse effects on final adult height, weight, or BMI. This may support the continued use of short courses in appropriate clinical scenarios, particularly where the benefits outweigh the potential risks. Evidence and guidelines do not support the use of systemic steroids for acute virus respiratory tract infections, but such a practice appears to have been common during the previous decade, whereas recent practice focuses on the identification of specific wheezing phenotypes which might benefit from systemic corticosteroid treatment.

This study is not without limitations. While the relatively small size of the ssccINF group enhanced the authenticity of our cohort, it may limit the generalizability of the findings. A larger sample size could provide more robust data and help to clarify any subtle effects of systemic steroid use on growth outcomes. Future studies with larger and more diverse cohorts are needed to validate these findings and explore potential variations based on factors such as dosage, duration of treatment, and underlying health conditions or to assess other potential outcomes such as bone density, hypothalamic–pituitary–adrenal axis, lung function, or cognitive development.

## 5. Conclusions

The use of short-course systemic steroids in infancy remains an essential therapeutic tool, provided that clinicians are aware of the potential risks and monitor growth and bone health appropriately. The findings of this study uniquely contribute to the growing body of evidence that short-term exposure to systemic steroids for few days during infancy does not have a significant long-term impact on final adult height, weight, and BMI making it a safe and effective option in the management of respiratory diseases in infants. Future research could further investigate potential sex-specific responses and evaluate additional health outcomes to refine guidelines on systemic steroid use in early childhood.

## Figures and Tables

**Table 1:** The demographic and clinical characteristics of the groups. A comparison between the included and excluded subjects is presented.

Variable (%)	Included Cases *n* = 257				Excluded Cases *n* = 38	*p*-Value 95% CI
	Controls 164 (63.8%)	ssccINF 23 (8.9%)	*p*-Value 95%CI	All Groups		
Male	75 (45.7)	12 (52.2)	0.65 0.85–1.08	127 (49.4)	28 (73.7)	0.005 0.88–0.96
Age at entry (y)	11.29 ± 3.5	10.35 ± 3.6	0.24 −0.72–2.58	11.2 ± 3.5	10.2 ± 3.7	0.12 −2.2–0.33
Cesarean birth	52 (31.7)	11 (47.8)	0.24 0.27–1.26	91 (35.4)	11 (28.9)	0.84 0.58–2.26
Breast feeding	107 (65.2)	16 (69.5)	0.82 0.91–1.15	166 (64.6)	24 (63.1)	0.85 0.58–1.98
Food allergy	17 (10.3)	1 (4.3)	0.70 0.81–1.04	27 (10.5)	3 (7.9)	0.78 0.46–4.2
Ever asthma diagnosed	63 (38.4)	13 (56.5)	0.11 0.24–1.13	134 (52.1)	17 (44.7)	0.30 0.79–2.54
Hospitalization (any)	41 (25)	12 (52.2)	0.02 0.19–0.85	84 (32.9)	10 (26.3)	0.35 0.71–1.04
Hospitalization for acute respiratory disease	10 (6.1)	6 (26.1)	0.006 0.09–0.58	33 (12.8)	2 (5.3)	0.28 0.61–1.61
Fracture	26 (15.8)	2 (8.7)	0.53 0.83–1.05	38 (14.8)	12 (31.5)	0.09 0.27–1.04
Age fracture occurred (y)	9.2 ± 3.8	11.5 ± 9.2	0.47 −8.5–4.08	8.9 ± 4.1	9.2 ± 3.9	0.78 −2.4–3.18
Exercise	143 (87.2)	19 (82.6)	0.88 0.81–1.05	219 (85.2)	30 (78.9)	0.71 0.56–3.1
Day/week exercise	2.6 ± 0.8	2.8 ± 1	0.35 −0.07–0.26	2.6 ± 0.9	2.5 ± 0.9	0.5 0.42–1.20
Family history of allergy	83 (50.6)	15 (65.2)	0.27 0.95–1.2	140 (54.5)	14 (36.8)	0.03 1.06–3.6
Smoking in family	70 (42.7)	7 (30.4)	0.26 0.84–1.04	104 (40.4)	24 (63.1)	0.01 0.21–0.75
Height at entry	149.2 ± 19.7	142.2 ± 20.6	0.11 −1.86–16.8	148.5 ± 19.6	145.3 ± 22.4	0.35 −9.8–3.5
BMI at entry	19.3 ± 3.9	18.4 ± 2.5	0.13 −0.3–2.15	19.4 ± 3.7	19.2 ± 4.6	0.88 −1.2–1.4
Tanner II at entry	37 (22.5)	4 (17.4)	0.71 0.82–1.03	59 (22.9)	8 (21.0)	0.33 0.72–1.06

**Table 2:** Multiple linear regression model evaluating the influence of various clinical factors on final height, weight, and BMI.

	Final Height		Final Weight			Final BMI
	*p*	95.% CI Lower, Upper	*p*	95% CI Lower, Upper	*p*	95% CI Lower, Upper
Sex	0.001	−16.47, −12.55	0.001	−17.29, −10.47	0.001	−2.00, 0.26
Cumulative days of systemic steroids	0.19	−1.00, 0.21	0.72	−1.24, 0.87	0.80	−0.30, 0.39
Age at entry (y)	0.22	−0.10, 0.44	0.05	−0.03, 0.90	0.15	−0.40, 0.26
Fracture	0.75	−3.17, 2.30	0.24	−1.92, 7.53	0.27	−0.70, 2.44
Breast feeding	0.67	−1.60, 2.47	0.11	−6.43, 0.65	0.06	−2.29, 0.06
Food allergy	0.06	−0.83, 0.35	0.14	−1.39, 9.63	0.71	−1.49, 2.17
Ever asthma diagnosed	0.61	−1.44, 2.44	0.23	−1.33, 5.42	0.25	−0.47, 1.77
Smoking in family	0.24	−3.18, 0.82	0.20	−1.21, 5.71	0.06	−0.47, 1.77
Exercise	0.97	−1.11, 1.08	0.65	−1.46, 2.33	0.74	−0.47, 1.77

## Data Availability

Data are available upon demand.
